# Acupuncture and related therapies for tension-type headache: a systematic review and network meta-analysis

**DOI:** 10.3389/fneur.2023.1194441

**Published:** 2023-06-22

**Authors:** Jinglin Hu, Xichen Wang, Siqi Jia, Lingzu Kong, Yining Wang, Xiaoqi Xin, Yinghua Hu, Xinhua Chen

**Affiliations:** ^1^College of Acupuncture and Tuina, Changchun University of Chinese Medicine, Changchun, Jilin, China; ^2^School of Medical Information, Changchun University of Chinese Medicine, Changchun, Jilin, China; ^3^Teaching and Research Office of Acupuncture and Tuina, Changchun University of Traditional Chinese Medicine, Changchun, Jilin, China; ^4^The Affiliated Hospital of Changchun University of Traditional Chinese Medicine, Changchun, Jilin, China

**Keywords:** acupuncture, tension-type headache, systematic review, randomized controlled trials, network meta-analysis

## Abstract

**Background:**

Tension-type headache (TTH) is one of the most common primary headaches. Several studies have confirmed the efficacy of acupuncture therapies for TTH, but it is uncertain which treatment is most effective.

**Objective:**

This study aimed to compare the effectiveness and safety of different acupuncture therapies for TTH using Bayesian Network Meta-analysis to provide new ideas for treating TTH.

**Methods:**

Nine databases were searched for randomized controlled trials (RCTs) about different acupuncture therapies for TTH up to December 1, 2022. The outcome indicators analyzed in our study were total effective rate, visual analog scale (VAS), headache frequency, and safety. Pairwise meta-analysis and risk of bias assessment were performed using Review Manager 5.4. Stata 15.0 generated a network evidence plot and detected publication bias. Finally, a Bayesian network meta-analysis of the data was used by RStudio.

**Results:**

The screening process resulted in 30 RCTs that met the inclusion criteria, including 2,722 patients. Most studies failed to report details of trials and were therefore assessed as unclear risks. Two studies were considered high risk because they did not report on all pre-specified outcome indicators or had incomplete data on outcome indicators. The NMA results showed that for total effective rate, bloodletting therapy had the most considerable SUCRA value (0.93156136), for VAS, head acupuncture combined with Western medicine ranked first (SUCRA = 0.89523571), and acupuncture combined with herbal medicine was the most effective in improving headache frequency (*p* > 0.05).

**Conclusion:**

Acupuncture can be used as one of the complementary or alternative therapies for TTH; bloodletting therapy better improves the overall symptoms of TTH, head acupuncture combined with Western medicine is more effective in reducing VAS scores, and acupuncture combined with herbal medicine seems to reduce headache frequency, but the difference is not statistically significant. Overall, acupuncture for TTH is effective with mild side effects, but future high-quality studies are still necessary.

**Systematic review registration:**

https://www.crd.york.ac.uk/prospero/, PROSPERO [CRD42022368749].

## 1. Introduction

Headache, a common neurological condition, has the second highest prevalence of non-fatal diseases worldwide. TTH is one of the most common primary headaches and is typically characterized by bilateral, compressive, or constricting headaches, often accompanied by photophobia and phonophobia (1). According to the latest epidemiological survey of the International Headache Society, the lifetime prevalence of TTH ranges from 30 to 78% and is influenced by different cultures, regions, genders, and ages (2, 3). TTH stems from mental or physical stress, sleep disorders, irregular diet, lack of physical activity, and menstrual cycle or hormonal changes in women. When TTH gradually becomes chronic, it can seriously affect people's daily activities, quality of life, productivity, and socioeconomics. In 2010, the European Federation of Neurological Societies (EFNS) published guidelines for the treatment of TTH, stating that simple analgesics and non-steroidal anti-inflammatory drugs (NSAIDs) are commonly used to treat episodic tension-type headache (ETTH). The tricyclic antidepressant amitriptyline is the drug of choice for preventing chronic tension-type headache (CTTH). Still, frequent and excessive use should be avoided due to gastrointestinal and other side effects (4). Because of the range of limitations of pharmacotherapy, there is value in exploring non-pharmacological therapies (4, 5).

As part of traditional Chinese medicine, acupuncture has been shown in previous studies to have significant benefits in treating pain (6). Its analgesic mechanisms are related to central, peripheral, spinal, and supraspinal analgesic mechanisms (7–10). In addition, there is evidence that acupuncture can reduce the degree of headache, decrease the frequency of headaches, reduce the dose of medication taken, and improve the quality of life. Various acupuncture methods for treating TTH include conventional acupuncture, electroacupuncture, head acupuncture, bloodletting, warm needle acupuncture, and other therapies (11–13). Still, there need to be more studies comparing the effectiveness and safety of different acupuncture types for treating TTH. It is unclear which acupuncture therapy is more effective for treating TTH.

Based on these uncertainties, we chose Network Meta-Analysis (NMA) (14) aimed at comparing the effectiveness of different acupuncture types for treating TTH and providing the best treatment plan for the clinical treatment of TTH with acupuncture.

## 2. Materials and methods

This study has been registered on the PROSPERO platform (https://www.crd.york.ac.uk/prospero/) Registration number: CRD42022368749. Furthermore, this study followed the Preferred Reporting Items for Systematic Reviews and Meta-Analyses for Network Meta-Analysis (PRISMA-NMA) checklist (Supplementary Appendix 1).

### 2.1. Search strategies

The following databases were searched for RCTs of acupuncture therapy for TTH until December 1, 2022: Cochrane Library, PubMed, Web of Science, EMBASE, China Knowledge Network (CNKI), Wanfang datebase, Vip, China Biomedical Database (CBM), and PROSPERO. The detailed search terms and search strategies are shown in Table 1.

**Table 1:** Search strategies.

**Database**	**Search term**
PubMed	#1 “Tension-Type Headache” [Mesh]
	#2 Tension Type Headache [Title/Abstract] OR Tension-Type Headaches [Title/Abstract] OR Headache, Tension-Type [Title/Abstract] OR Tension-Type [Title/Abstract] OR Stress Headache [Title/Abstract] OR Headache, Stress [Title/Abstract] OR Headaches, Stress [Title/Abstract] OR Stress Headaches [Title/Abstract] OR Tension Headache [Title/Abstract] OR Headache, Tension [Title/Abstract] OR Headaches, Tension [Title/Abstract] OR Tension Headaches [Title/Abstract] OR Tension-Vascular Headache [Title/Abstract] OR Headache, Tension-Vascular [Title/Abstract] OR Headaches, Tension-Vascular [Title/Abstract] OR Tension Vascular Headache [Title/Abstract] OR Tension-Vascular Headaches [Title/Abstract]
	#3 #1 OR #2
	#4 “Acupuncture” [Mesh]
	#5 “Acupuncture Therapy” [Mesh]
	#6 “Acupuncture Points” [Mesh]
	#7 “Acupuncture Analgesia” [Mesh]
	#8 Acupuncture [Title/Abstract] OR Electroacupuncture [Title/Abstract] OR Electro-acupuncture [Title/Abstract] OR Acupuncture Analgesia [Title/Abstract] OR Acupuncture Treatment [Title/Abstract] OR Acupuncture Treatments [Title/Abstract] OR Treatment, Acupuncture [Title/Abstract] OR Therapy, Acupuncture [Title/Abstract] OR Tigger point [Title/Abstract] OR Acupuncture Point [Title/Abstract] OR Point, Acupuncture [Title/Abstract] OR Points, Acupuncture [Title/Abstract]
	#9 #4 OR #5 OR #6 OR #7 OR #8
	#10 “Randomized Controlled Trial” [Publication Type]
	#11 “Randomized Controlled Trials as Topic” [Mesh]
	#12 “Pragmatic Clinical Trial” [Publication Type]
	#13 “Pragmatic Clinical Trials as Topic” [Mesh]
	#14 “Intention to Treat Analysis” [Mesh]
	#15 “random allocation' [Mesh Terms]
	#16 #10 OR #11 OR #12 OR #13 OR #14 OR #15
	#17 #3 AND #9 AND #16
Cochrane library	#1 MeSH descriptor: [Tension-Type Headache] explode all trees
	#2 ‘Tension Type Headache':ti,ab OR ‘Tension-Type Headaches':ti,ab OR ‘Headache, Tension-Type':ti,ab OR 'Tension-Type':ti,ab OR ‘Stress Headache':ti,ab OR 'Headache, Stress':ti,ab OR 'Headaches, Stress':ti,ab OR ‘Stress Headaches':ti,ab OR ‘Tension Headache':ti,ab OR 'Headache, Tension':ti,ab OR 'Headaches, Tension':ti,ab OR ‘ Tension Headaches':ti,ab OR 'Tension-Vascular Headache':ti,ab OR 'Headache, Tension-Vascular':ti,ab OR 'Headaches,Tension-Vascular':ti,ab OR ‘Tension Vascular Headache':ti,ab OR 'Tension-Vascular Headaches':ti,ab
	#3 #1 OR #2
	#4 MeSH descriptor: [Acupuncture] explode all trees
	#5 MeSH descriptor: [Acupuncture Therapy] explode all trees
	#6 MeSH descriptor: [Acupuncture Analgesia] explode all trees
	#7 ‘acupuncture':ti,ab OR ‘acupuncture analgesia':ti,ab OR ‘trigger point':ti,ab OR ‘electro-acupuncture':ti,ab OR ‘Acupuncture Treatment':ti,ab OR ‘Acupuncture Treatments':ti,ab OR ‘Treatment, Acupuncture ‘:ti,ab OR ‘Therapy,Acupuncture':ti,ab OR ‘Tigger point':ti,ab OR ‘Acupuncture Point':ti,ab OR ‘Point, Acupuncture':ti,ab OR ‘Points, Acupuncture':ti,ab
	#8 #4 OR #5 OR #6 OR #7
	#9 ‘randomized controlled trial'/exp
	#10 ‘randomized controlled trial'/exp
	#11 #9 OR #10
	#12 #3 AND #8 AND #11
Web of science	#1 TS = (Tension-type headache OR Tension Type Headache OR Tension-Type Headaches OR Headache, Tension-Type OR Tension-Type OR Headache, Stress OR Headaches, Stress OR Stress Headaches OR Headache, Tension OR Headaches, Tension OR Tension Headaches OR Tension-Vascular Headache OR Headache, Tension-Vascular OR Headaches, Tension-Vascular OR Tension Vascular Headache OR Tension-Vascular Headaches
	#2 TS = (Acupuncture OR Acupuncture Therapy OR Therapy, Acupuncture OR Acupuncture Points OR Point, Acupuncture OR OR Points, Acupuncture OR Acupuncture Analgesia OR trigger point OR electroacupuncture OR electro-acupuncture OR Acupuncture Treatment OR Treatment, Acupuncture)
	#3 TS = (Randomized Controlled Trial OR Randomized Controlled Trials as Topic OR Random)
	#4 #1 AND #2 AND #3
Embase	#1 ‘Tension-type headache'/exp
	#2 ‘Tension Type Headache':ti,ab OR ‘Tension-Type Headaches':ti,ab OR ‘Headache, Tension-Type':ti,ab OR ‘Tension-Type':ti,ab OR ‘Stress Headache':ti,ab OR ‘Headache, Stress':ti,ab OR ‘Headaches, Stress':ti,ab OR ‘Stress Headaches':ti,ab OR ‘Tension Headache':ti,ab OR ‘Headache, Tension':ti,ab OR ‘Headaches, Tension':ti,ab OR ‘ Tension Headaches':ti,ab OR ‘Tension-Vascular Headache':ti,ab OR ‘Headache, Tension-Vascular':ti,ab OR ‘Headaches,Tension-Vascular':ti,ab OR ‘Tension Vascular Headache':ti,ab OR ‘Tension-Vascular Headaches':ti,ab
	#3 #1 OR #2
	#4 ‘acupuncture'/exp
	#5 ‘acupuncture analgesia'/exp
	#6 ‘electroacupuncture'/exp
	#7 ‘acupuncture':ti,ab OR ‘acupuncture needle':ti,ab OR ‘trigger point':ti,ab OR ‘electro-acupuncture':ti,ab OR ‘Acupuncture Treatment':ti,ab OR ‘Acupuncture Treatments':ti,ab OR ‘Treatment, Acupuncture ‘:ti,ab OR ‘Therapy,Acupuncture':ti,ab OR ‘Tigger point':ti,ab OR ‘Acupuncture Point':ti,ab OR ‘Point, Acupuncture':ti,ab OR ‘Points, Acupuncture':ti,ab
	#8 #4 OR #5 OR #6 OR #7
	#9 ‘randomized controlled trial'/exp
	#10 ‘randomized controlled trial'/exp
	#11 #9 OR #10
	#12 #3 AND #8 AND #11
CNKI	#1 (主题 = 紧张型头痛 或者 题名 = 紧张型头痛 或者 v_subject = 中英文扩展 (紧张型头痛) 或者 title = 中英文扩展 (紧张型头痛)) (模糊匹配)
	#2 (主题 = 压力性头痛 或者 题名 = 压力性头痛 或者 v_subject = 中英文扩展 (压力性头痛) 或者 title = 中英文扩展 (压力性头痛)) (模糊匹配)
	#3 (主题 = 紧张性头痛 或者 题名 = 紧张性头痛 或者 v_subject = 中英文扩展 (紧张性头痛) 或者 title = 中英文扩展 (紧张性头痛)) (模糊匹配)
	#4 #1 OR #2 OR #3
	#5 (主题 = 针刺 或者 题名 = 针刺 或者 v_subject = 中英文扩展 (针刺) 或者 title = 中英文扩展 (针刺)) (模糊匹配)
	#6 (主题 = 针灸 或者 题名 = 针灸 或者 v_subject = 中英文扩展 (针灸) 或者 title = 中英文扩展 (针灸)) (模糊匹配)
	#7 (主题 = 电针 或者 题名 = 电针 或者 v_subject = 中英文扩展 (电针) 或者 title = 中英文扩展 (电针)) (模糊匹配)
	#8 (主题 = 头针 或者 题名 = 头针 或者 v_subject = 中英文扩展 (头针) 或者 title = 中英文扩展 (头针)) (模糊匹配)
	#9 (主题 = 针 或者 题名 = 针 或者 v_subject = 中英文扩展 (针)或者 title = 中英文扩展 (针)) (模糊匹配)
	#10 #4 OR #5 OR #6 OR #7 OR #8 OR #9
	#11 (摘要 = 随机 或者 abstract_en = 中英文扩展 (随机)) (模糊匹配)
	#12 (摘要 = RCT 或者 abstract_en = 中英文扩展 (RCT)) (模糊匹配)
	#13 #11 OR #12
	#14 #4 AND #10 AND #13
VIP	((((题名或关键词 = 紧张型头痛 OR 题名或关键词 = 压力性头痛) OR 题名或关键词 = 头痛) AND ((((题名或关键词 = 针刺 OR 题名或关键词 = 针灸) OR 题名或关键词 = 电针) OR 题名或关键词 = 头针) OR 题名或关键词 = 针)) AND (文摘 = 随机 OR 文摘 = RCT))
WanFang	主题 :(紧张型头痛 OR 压力性头痛 OR 头痛) and 主题 :(针刺 OR 针灸 OR 电针 OR 针) and 主题 :(随机 OR RCT)

### 2.2. Eligibility criteria

(1) Study type: Randomized Controlled Trials (RCTs) of acupuncture for TTH, with language restricted to Chinese and English.
(2) Participants: Meet the diagnostic criteria for tension-type headache in the third edition of the headache classification published by the International Headache Society (IHS) (3), with no restrictions on nationality, age, gender, or culture.
(3) Interventions and comparisons: The interventions in the treatment group were acupuncture-related therapy (conventional acupuncture, head acupuncture, electroacupuncture, bloodletting therapy, etc.) or acupuncture combined with other therapies (Western medicine, Chinese medicine, etc.), and in the control group, another treatment of acupuncture, pharmacotherapy (Western medicine, herbal medicine)—no restrictions on acupuncture's time, depth, or duration.
(4) Outcome indicators: The outcome indicators must include at least one of the following, total effective rate, VAS, headache frequency, etc. Also, the occurrence of adverse events will be recorded.
(5) Exclusion criteria: duplicate studies; case reports; animal experiments; literature reviews; protocol; meta-analysis; dissertations.

### 2.3. Study selection

We searched literature according to the above inclusion criteria and imported all the searched literature into EndNote X9. Two independent researchers (YW and XX) performed the screening independently. We first excluded duplicates and read the titles and abstracts of the remaining. After excluding some of the literature, the remaining was selected for inclusion and exclusion after reading the full text. A third researcher will discuss and resolve disagreements arising during this process (JH).

### 2.4. Data extraction

Two independent researchers (YW and XX) performed data extraction of the final included literature and recorded it in Microsoft Excel 2016. The main extracts had: first author, study country, year of publication, intervention, case characteristics, sample size, outcome indicators, adverse events, etc. Any disagreement will be resolved in consultation with the third investigator (JH).

### 2.5. Quality assessment

Two independent researchers (SJ and LK) evaluated the risk of bias in the included studies using Review Manager 5.4, a risk of bias assessment tool recommended by the Cochrane Handbook (15). The tool assesses the risk of bias of RCTs in seven aspects, random sequence generation, allocation hiding, blinding of participating researchers, blinding of outcome assessment, incomplete outcome data, selective reporting, and other biases. The results of the risk of bias assessment are assessed by high risk, low risk, and unclear risk. Two researchers will independently evaluate, and disagreements will be negotiated and agreed upon with a third investigator (JH).

### 2.6. Statistical analysis

First, a pairwise meta-analysis was performed using Review Manager 5.4. The odds ratio (OR) and 95% Confidence Interval (CI) were used to analyze binary variables, and the mean difference (MD) was used to analyze continuous variables. Cochran's *I*-square (*I*^2^) was used to determine heterogeneity, and a fixed-effects model was selected for data analysis with *I*^2^ < 50% and the random effects model with *I*^2^ > 50%. For OR and MD, *p* < 0.05 represented statistical significance.

To compare the effectiveness of different acupuncture therapies for TTH, we performed an NMA using Stata 15.0 and RStudio software. The “gemtc” and “JAGs-4.3.1” packages in RStudio were used for data analysis. Binary variables were analyzed using the odds ratio (OR) as the effect size. Continuous variables were analyzed using mean difference (MD). 95% CI was calculated for the effect sizes, with 95% CI not containing one being considered statistically significant; the “mvmeta” package in Stata was used to generate a network evidence plot, where each node represents an intervention, the size of the node represents the sample size, the line between two nodes represents the existence of a direct comparison between two interventions and the thickness of the line represents the sample size of the intervention for which a direct comparison exists. When there is a closed loop, the node splitting method is required to compare the inconsistency between the results of indirect comparisons and direct comparisons, and the consistency model is tested based on the Markov chain-Monte Carlo method (MCMC) framework, the consistency model will be used when *p* > 0.05; the potential scale reduction factor (PSRF) was used to assess the convergence effect of the NMA results when the PSRF value is closer to 1, it indicates that the convergence result is better and the results are reliable; for the final results, we generated a two-by-two comparison table. The surface under the cumulative ranking (SUCRA) presented a ranking of the interventions, with larger SUCRA values indicating a higher probability of being the best intervention. In addition, we also generated histograms of ranking probabilities. Finally, we used Stata to generate comparative corrected funnel plots to test for publication bias and small sample effects.

## 3. Results

### 3.1. Literature search

**Figure 1:**
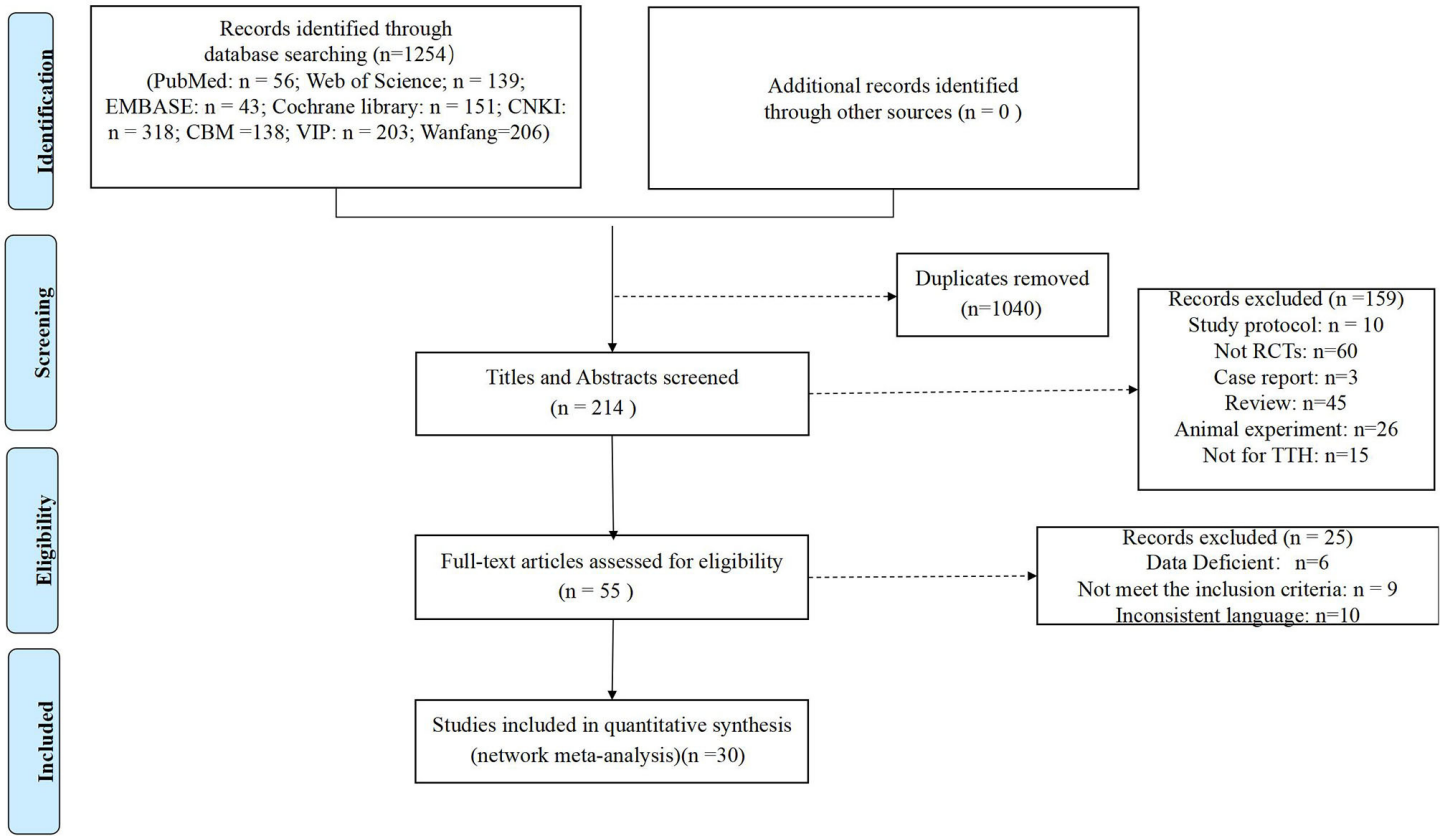
Flow chart of study selection.

We searched 1,254 studies according to the pre-developed search strategies, and 30 RCTs (16–45) were finally included in this study. The detailed screening process of the literature is shown in Figure 1.

### 3.2. Characteristics of the included studies

The basic characteristics of the 30 RCTs are shown in Table 2. Thirty RCTs were published from 2000 to 2022. All trials were conducted in China and included a total of 2,722 patients (aged 18–65 years) with TTH. Thirty RCTs had one three-arm trial (23) and 29 two-arm trials; interventions in the treatment group included conventional acupuncture, head acupuncture, Plum Blossom Needle Tapping, bloodletting therapy, and electroacupuncture; interventions in the control group included another acupuncture therapy, Western medicine, or herbal medicine. The above treatments could be used alone or in combination. Each RCT used the VAS score or total effective rate (or a combination) to determine the efficacy.

**Table 2:** Characteristics of the included studies.

**References**	**Country**	**Invention**	**Control**					
		**Treatment**	***n***	**Age (mean** ±**sd)/range**	**Treatment**	***n***	**Age (mean** ±**sd)/range**	**Outcome**
Wang et al. (39)	China	Acu	150	44 ± 13	WM	100	43 ± 13	
Luo et al. (31)	China	Acu	42	45.31 ± 7.58	WM	43	45.13 ± 8.12	
Guo and Shen (23)	China	HA	50	33.9 ± 10.2	Acu/WM	50/50	33.2 ± 10.2/34.0 ± 10.6	
Jiang (24)	China	Acu+HM	40	39.7 ± 4.3	WM	40	38.5 ± 4.8	
Wang et al. (38)	China	Acu	50	37.64 ± 3.42	WM	50	35.17 ± 4.03	
Zhou (44)	China	Acu+WM	34	48.21 ± 3.80	WM	34	47.62 ± 3.11	
Gui et al. (20)	China	HA+WM	30	38 ± 13	WM	30	39 ± 11	
Wang et al. (37)	China	Acu	29	38 ± 10	WM	27	39 ± 11	
Chen and Feng (18)	China	Acu	70	39.89 ± 9.48	WM	70	40.36 ± 9.48	
Zhang (43)	China	Acu+HM	38	39.3 ± 5.1	WM	38	39.7 ± 5.2	
Zou et al. (45)	China	Acu+BT	30	36.54 ± 11.60	Acu	30	37.93 ± 11.80	
Li (29)	China	Acu	60	38.5 ± 2.7	WM	60	36.6 ± 2.9	
Jin et al. (25)	China	BT	30	45.3 ± 3.9	WM	30	43.2 ± 3.2	
Wu and Sun (40)	China	Acu+HM	30	18–60	WM	30	17–61	
Guo (22)	China	Acu+HM	40	36.5 ± 3.2	WM	40	38.7 ± 3.8	
Shi (34)	China	Acu+HM	45	36 ± 10.5	WM	48	41 ± 8.5	
Duan (19)	China	Acu	48	42.7 ± 11.5	WM	45	43.5 ± 11.2	
Chen (17)	China	Acu	34	32.6 ± 4.5	HM	32	37.2 ± 6.2	
Sun and Guo (35)	China	PBNT	30	43.5 ± 11.7	WM	30	42.8 ± 11.3	
Liu and Liu (30)	China	Acu+HM	91	18–66	HM	31	19–64	
Li and Liu (28)	China	Acu	40	18–60	HM	40	18–60	
Tong et al. (36)	China	Acu+HM	43	43.4 ± 11.2	WM	43	42.7 ± 10.4	
Chen et al. (16)	China	BT	45	21–67	Acu	45	18–64	
Peng (33)	China	Acu	63	32.5 ± 5.6	WM	63	31.3 ± 6.3	
Zhang et al. (42)	China	Acu	26	18–65	WM	20	18–65	
Guo (21)	China	BT	30	26.5 ± 15.2	WM	30	24.6 ± 15.4	
Li et al. (27)	China	Acu	30	20–30	WM	25	20–30	
Kang (26)	China	Acu+HM	58	19–48	HM	33	20–40	
Peng (32)	China	EA+CT	82	36.23 ± 6.25	WM	81	34.68 ± 5.27	
Xu (41)	China	EA	76	26–61	WM	70	28–63	

, Visual Analog Scale, VAS;, total effective rate;, frequency of headache;, adverse events. HA, head acupuncture; Acu, acupuncture; EA, electro-acupuncture; HM, herbal medicine; BT, bloodletting therapy; CT, cupping therapy; PBNT, Plum Blossom Needle Tapping; WM, Western medicine.

### 3.3. Quality evaluation of included literatures

**Figure 2:**
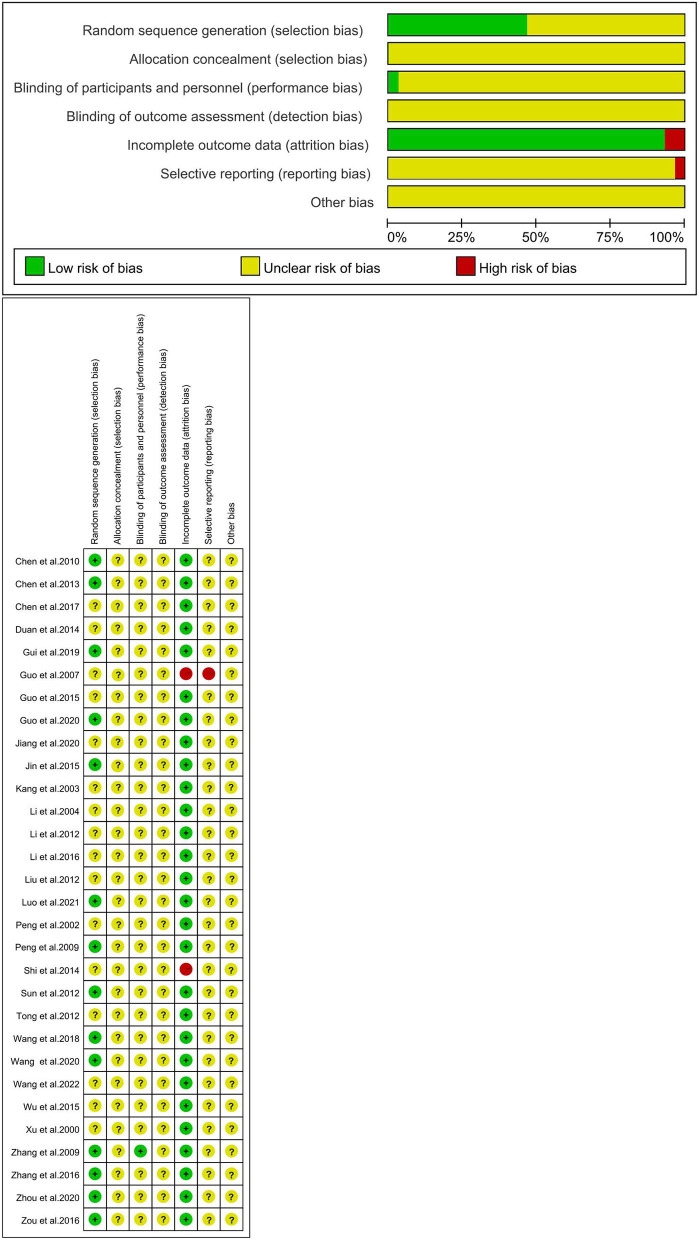
Study quality assessment.

Fourteen studies (16, 17, 20, 23, 25, 31, 33, 35, 37, 38, 42–45) used the random number table method and were assessed as low risk; we classified all studies as an unclear risk because they did not mention the allocation concealment method; one study (42) described in detail the use of blinding of subjects and was rated as low risk; all studies did not mention blinding of outcome assessors and were rated as an unclear risk; two studies (21, 34) that did not report all outcomes or had incomplete data were classified as high risk; one study (21) was a high risk of selective reporting bias because it did not report all of the prespecified outcome indicators, and all other studies were difficult to determine and were rated as unclear risk (Figure 2).

### 3.4. Pairwise meta-analysis

We performed 13 pairwise meta-analyses to compare the improvement of overall symptoms of TTH with different acupuncture therapies (Table 3). Compared with Western medicine, conventional acupuncture (10 RCTs, OR = 4.37, 95% CI = 2.85–6.59, *I*^2^ = 0%, *p* < 0.00001), acupuncture + Western medicine (1 RCT, 0R = 7.07, 95% CI = 1.80–62.31, *p* = 0.03), acupuncture + herbal medicine (6 RCTs, OR = 3.92, 95% CI = 2.18–7.04, *I*^2^ = 0%, *p* < 0.00001), electroacupuncture + cupping (1RCT, OR = 4.14, 95% CI = 1.11–15.44, *p* = 0.03), head acupuncture (1RCT, OR = 6.00, 95% CI = 1.24–28.99), head acupuncture + Western medicine (1RCT, OR = 7.00, 95% CI = 1.38–35.48, *p* = 0.02), and Plum Blossom Needle Tapping (1RCT, OR = 3.76, 95% CI = 1.04–13.65, *p* = 0.04) were more effective in improving the total effective rate of TTH; compared with herbal medicine, conventional acupuncture (2RCTs, OR = 5.09, 95% CI = 2.08–12.50, *I*^2^ = 77%, *p* = 0.0003) and acupuncture + herbal medicine (2RCTs, OR = 6.85, 95% CI = 2.46–19.08, *I*^2^ = 0%, *p* = 0.0002) were more effective; moreover, bloodletting therapy (1RCT, OR = 6.32, 95% CI = 1.67–23.92, *p* = 0.007) was more effective than conventional acupuncture. However, there was no statistically significant difference in electroacupuncture vs. Western medicine, acupuncture + bloodletting vs. conventional acupuncture, and head acupuncture vs. conventional acupuncture.

**Table 3:** Pairwise meta-analysis of total effective rate.

**Intervention**	**Number**	**OR (95% CI)**	***I*^2^ (%)**	***P***
Acu + HM vs. WM	6	**3.92 (2.18, 7.04)**	0	*P* < 0.00001
Acu + WM vs. WM	1	**7.07 (1.80, 62.31)**	–	0.03
Acu vs. WM	10	**4.37 (2.89, 6.59)**	0	*P* < 0.00001
EA + CT vs. WM	1	**4.14 (1.11, 15.44)**	–	0.03
EA vs. WM	1	1.01 (0.44, 2.33)	–	0.98
HA + WM vs. WM	1	**7.00 (1.38, 35.48)**	–	0.02
HA vs. WM	1	**6.00 (1.24, 28.99)**	–	0.03
PBNT vs. WM	1	**3.76 (1.04, 13.65)**	–	0.04
Acu + HM vs. HM	2	**6.85 (2.46, 19.08)**	0	0.0002
Acu + BT vs. Acu	1	2.15 (0.36, 12.76)	–	0.40
Acu vs. HM	2	**5.09 (2.08, 12.50)**	77	0.0004
BT vs. Acu	1	**6.32 (1.67, 23.92)**	–	0.007
HA vs. Acu	1	1.53 (0.24, 9.59)	–	0.65

The bold font indicates a statistical difference. HA, head acupuncture; Acu, acupuncture; EA, electro-acupuncture; HM, herbal medicine; BT, bloodletting therapy; CT, cupping therapy; PBNT, Plum Blossom Needle Tapping; WM, Western medicine.

In terms of VAS scores (Table 4), conventional acupuncture (4RCTs, MD = 1.48, 95% CI = 1.36–1.60, *I*^2^ = 96%, *p* < 0.00001), acupuncture + Western medicine (1RCT, MD = 2.20, 95% CI = 1.86–2.54, *p* < 0.00001), and head acupuncture + Western medicine (1RCT, MD = 2.30, 95% CI = 1.67–2.93, *p* < 0.00001) were more effective than Western medicine; in addition, conventional acupuncture (1RCT, MD = 2.25, 95% CI = 1.76–2.74, *p* < 0.00001) was more effective than herbal medicine. There was no statistically significant difference when comparing acupuncture + herbal medicine with Western medicine, bloodletting therapy with acupuncture, bloodletting therapy with Western medicine, or Plum Blossom Needle Tapping with Western medicine.

**Table 4:** Pairwise meta-analysis of VAS.

**Intervention**	**Number**	**MD (95% CI)**	***I*^2^ (%)**	***P***
Acu + HM vs. WM	2	0.71 (0.09, 1.33)	83	0.06
Acu + WM vs. WM	1	**2.20 (1.86, 2.54)**	–	*P* < 0.00001
Acu vs. HM	1	**2.25 (1.76, 2.74)**	–	*P* < 0.00001
Acu vs. WM	4	**1.48 (1.36, 1.60)**	96	*P* < 0.00001
BT vs. Acu	1	**0.03 (-0.22, 0.16)**	–	0.76
BT vs. WM	1	0.95 (0.60, 1.30)	–	*P* < 0.00001
HA + WM vs. WM	1	**2.30 (1.67, 2.93)**	–	*P* < 0.00001
PBNT vs. WM	1	0.82 (0.26, 1.38)	–	0.004

The bold font indicates a statistical difference. HA, head acupuncture; Acu, acupuncture; HM, herbal medicine; BT, bloodletting therapy; PBNT, Plum Blossom Needle Tapping; WM, Western medicine.

For headache frequency, compared with Western medicine, acupuncture + Western medicine (1RCT, MD = 1.65, 95% CI = 1.23–2.07, *p* = 0.01) conventional acupuncture (2RCTs, MD = 0.24, 95% CI = 0.17–0.31, *I*^2^ = 0%, *p* < 0.00001), bloodletting therapy (1RCT, MD = 0.70, 95% CI = 0.57–0.83, *p* < 0.00001) all showed advantages (Table 5).

**Table 5:** Pairwise meta-analysis of headache frequency.

**Intervention**	**Number**	**MD (95% CI)**	***I*^2^ (%)**	***P***
Acu + HM vs. WM	1	1.70 (0.52, 2.88)	–	0.01
Acu + WM vs. WM	1	**1.65 (1.23, 2.07)**	–	0.88
Acu vs. WM	2	**0.24 (0.17, 0.31)**	0	*p* < 0.00001
BT vs. WM	1	**0.70 (0.57, 0.83)**	–	*p* < 0.00001
PBNT vs. WM	1	1.48 (0.38, 2.58)	–	–

The bold font indicates a statistical difference. Acu, acupuncture; HM, herbal medicine; BT, bloodletting therapy; PBNT, Plum Blossom Needle Tapping; WM, Western medicine.

### 3.5. Network meta-analysis

#### 3.5.1. Network evidence plot

**Figure 3:**
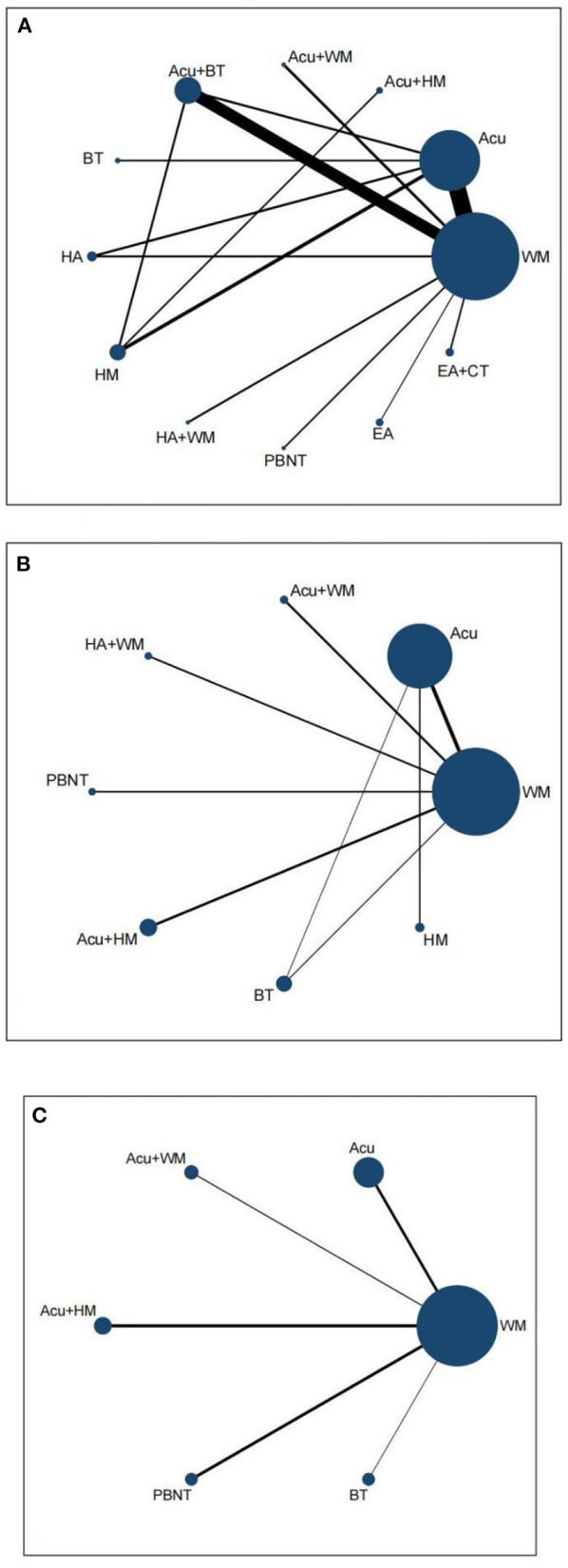
Network evidence plot. **(A)** Total effective rate. **(B)** VAS. **(C)** Frequency of headache. HA, head acupuncture; Acu, acupuncture; EA, electro-acupuncture; HM, herbal medicine; BT, bloodletting therapy; CT, cupping therapy; PBNT, Plum Blossom Needle Tapping; WM, Western medicine.

Data on the total effective rate of acupuncture therapies to improve TTH came from 27 RCTs (16–18, 20, 22–24, 26–45), including a total of 2,656 patients; 13 interventions included conventional acupuncture, acupuncture + herbal medicine, acupuncture + Western medicine, acupuncture + bloodletting therapy, bloodletting therapy, head acupuncture, head acupuncture + Western medicine, herbal medicine, electroacupuncture, electroacupuncture + cupping, Plum Blossom Needle Tapping, and Western medicine. Figure 3A shows that the sample size of Western medicine was the largest, and the frequency of comparison between conventional acupuncture and Western medicine was the highest. Data for VAS scores were obtained from 12 RCTs (16, 19, 20, 22, 25, 28, 35–39, 44) containing 1,086 patients and involving eight interventions which included Western medicine, acupuncture, acupuncture + Western medicine, head acupuncture + Western medicine, acupuncture + herbal medicine, Plum Blossom Needle Tapping, bloodletting therapy, and herbal medicine (Figure 3B). Data on the effect of acupuncture and its related therapies on headache frequency were from six RCTs (21, 35–38, 44), including 430 patients with six interventions, including Western medicine, conventional acupuncture, acupuncture + Western medicine, acupuncture + herbal medicine, Plum Blossom Needle Tapping, and bloodletting therapy (Figure 3C).

We used the node splitting method to test the inconsistency of the closed loops in the network evidence plot; *p* > 0.05 indicates no inconsistency, meaning that the results of direct and indirect comparisons are consistent (Supplementary Appendix 2). The model's fit was verified using the convergence diagnostic plot. The potential scale reduction factor (PSRF) tended to 1, suggesting a high degree of model convergence and reliable results of the NMA. The convergence diagnostic plots are shown in Supplementary Appendix 3.

#### 3.5.2. Network meta-analysis of total effective rate

The results of NMA showed that the total effective rate of conventional acupuncture, acupuncture + herbal medicine, acupuncture + Western medicine, acupuncture + bloodletting therapy, bloodletting therapy, head acupuncture, head acupuncture + Western medicine, electroacupuncture + cupping, and Plum Blossom Needle Tapping were all superior to Western medicine. In addition, bloodletting therapy was more effective than conventional acupuncture and acupuncture + herbal medicine (Table 6).

**Table 6:** Results of network meta-analysis for total effective rate.

**WM**											
**0.21 (0.14, 0.31)**	Acu										
**0.26 (0.13, 0.46)**	1.24 (0.6, 2.44)	Acu + HM									
**0.1 (0, 0.76)**	0.47 (0.02, 3.77)	0.37 (0.01, 3.62)	Acu + WM								
**0.09 (0.01, 0.61)**	0.42 (0.04, 2.8)	0.35 (0.03, 2.65)	0.92 (0.04, 37.26)	Acu + BT							
**0.03 (0.01, 0.13)**	**0.14 (0.03, 0.58)**	**0.11 (0.02, 0.6)**	0.3 (0.02, 9.51)	0.33 (0.03, 4.32)	BT						
**0.14 (0.02, 0.67)**	0.67 (0.08, 3.2)	0.54 (0.06, 2.74)	1.46 (0.06, 51.36)	1.57 (0.09, 27.73)	4.64 (0.38, 49.64)	HA					
2.32 (0.84, 6.82)	**10.98 (3.88, 33.21)**	**8.84 (3.46, 24.77)**	**23.71 (2.28, 689.7)**	**26.98 (2.92, 298.3)**	**79.68 (13.45, 573.4)**	**17 (2.59, 173.5)**	HM				
**0.13 (0.01, 0.66)**	0.61 (0.06, 3.25)	0.49 (0.04, 2.93)	1.33 (0.06, 44.01)	1.36 (0.07, 30.36)	4.21 (0.3, 47.96)	0.94 (0.06, 12.13)	**0.05 (0, 0.4)**	HA + WM			
**0.24 (1.05, 1.06)**	1.16 (0.24, 5.31)	0.93 (0.2, 4.57)	2.45 (0.17, 86.1)	2.75 (0.24, 40.24)	8.48 (0.83, 77.43)	1.73 (0.19, 22.76)	**0.1 (0.02, 0.6)**	1.86 (0.21, 30.23)	PBNT		
0.99 (0.34, 2.86)	**4.72 (1.49, 14.93)**	**3.79 (1.13, 13.25)**	10.02 (0.97, 285.3)	**11.14 (1.24, 137.2)**	**34.25 (5.37, 253.8)**	**7.13 (1.14, 78.01)**	0.43 (0.1, 1.83)	**7.71 (1.12, 101.1)**	4.05 (0.66, 25.96)	EA	
**0.22 (0.04, 0.89)**	1.03 (0.19, 4.48)	0.83 (0.15, 4.29)	2.14 (0.15, 74.75)	2.51 (0.18, 35.03)	7.24 (0.82, 75.32)	1.54 (0.16, 19.99)	**0.09 (0.01, 0.55)**	1.71 (0.17, 27.32)	0.88 (0.1, 7.54)	0.22 (0.03, 1.3)	EA + CT

The bold font indicates a statistical difference. HA, head acupuncture; Acu, acupuncture; EA, electro-acupuncture; HM, herbal medicine; BT, bloodletting therapy; CT, cupping therapy; PBNT, Plum Blossom Needle Tapping; WM, Western medicine.

The greater the SUCRA value, the higher ranking, proving the intervention is more effective. According to our findings, bloodletting therapy (SUCRA = 0.93156136) had the highest probability of being the best treatment to improve the overall symptoms of TTH, and herbal medicine (SUCRA = 0.01797955) had the lowest. The mean SUCRA values and the ranking of the 12 interventions are shown in Table 7, and the probability sorting histogram is shown in Supplementary Figure 1.

**Table 7:** SUCRA values of total effective rate and ranking of interventions.

**Rank**	**SUCRA**	**Treatments**
1	0.93156136	BT
2	0.74003636	Acu + BT
3	0.71301477	Acu + WM
4	0.66750909	HA + WM
5	0.64663750	HA
6	0.53312841	Acu
7	0.52475682	EA + CT
8	0.49331136	PBNT
9	0.44955909	Acu + HM
10	0.14442955	EA
11	0.13807614	WM
12	0.01797955	HM

HA, head acupuncture; Acu, acupuncture; EA, electro-acupuncture; HM, herbal medicine; BT, bloodletting therapy; CT, cupping therapy; PBNT, Plum Blossom Needle Tapping; WM, Western medicine.

#### 3.5.3. Network meta-analysis of VAS

The NMA results of VAS showed that conventional acupuncture, acupuncture + Western medicine, and head acupuncture + Western medicine were all the better than Western medicine for reducing VAS scores. Other comparisons were not statistically significant (Table 8).

**Table 8:** Results of network meta-analysis for VAS.

**WM**							
**0.37 (0.14, 0.98)**	Acu						
**0.11 (0.01, 0.81)**	0.3 (0.03, 2.7)	Acu + WM					
**0.1 (0.01, 0.69)**	0.27 (0.03, 2.4)	0.9 (0.06, 15.18)	HA + WM				
0.43 (0.06, 3.02)	1.18 (0.13, 10.48)	3.88 (0.24, 65.53)	4.31 (0.27, 72.49)	PBNT			
0.7 (0.17, 2.74)	1.91 (0.36, 9.83)	6.24 (0.57, 73.02)	6.94 (0.63, 77.45)	1.61 (0.15, 17.46)	Acu + HM		
0.38 (0.04, 3.29)	1.03 (0.15, 6.87)	3.38 (0.18, 67.25)	3.76 (0.21, 71.29)	0.87 (0.05, 16.98)	0.54 (0.05, 7.07)	BT	
3.48 (0.38, 30.25)	**9.43 (1.37, 67.8)**	**31.03 (1.71, 625.5)**	**34.45 (1.82, 708)**	8.02 (0.44, 152)	5.03 (0.4, 66.1)	9.11 (0.63, 136.5)	HM

The bold font indicates a statistical difference. HA, head acupuncture; Acu, acupuncture; HM, herbal medicine; BT, bloodletting therapy; PBNT, Plum Blossom Needle Tapping; WM, Western medicine.

Table 9 shows that the intervention with the most outstanding SUCRA value was head acupuncture + Western medicine. Rank order was head acupuncture + western medicine, acupuncture + Western medicine, conventional acupuncture, bloodletting therapy, Plum Blossom Needle Tapping, acupuncture + herbal medicine, Western medicine, and herbal medicine. The results suggest that head acupuncture combined with Western medicine may be the best intervention for reducing VAS scores. The probability sorting histogram is shown in Supplementary Figure 2.

**Table 9:** SUCRA values of VS and ranking of interventions.

**Rank**	**SUCRA**	**Treatments**
1	0.89523571	HA + WM
2	0.87895000	Acu + WM
3	0.58860714	Acu
4	0.56378571	BT
5	0.50989286	PBNT
6	0.35999286	Acu + HM
7	0.19531429	WM
8	0.02610714	HM

HA, head acupuncture; Acu, acupuncture; HM, herbal medicine; BT, bloodletting therapy; PBNT, Plum Blossom Needle Tapping; WM, Western medicine.

#### 3.5.4. Network meta-analysis of headache frequency

The NMA results for headache frequency are shown in Table 10. No statistically significant results were observed between comparisons of different acupuncture therapies.

**Table 10:** Results of network meta-analysis for headache frequency.

**WM**					
0.77 (0.21, 2.48)	Acu				
0.19 (0.04, 1.07)	0.25 (0.03, 2.26)	Acu + WM			
0.18 (0.03, 1.26)	0.24 (0.03, 2.32)	0.94 (0.08, 11.79)	Acu + HM		
0.23 (0.04, 1.56)	0.3 (0.03, 3.22)	1.19 (0.09, 15.08)	1.24 (0.09, 20.2)	PBNT	
0.5 (0.09, 2.63)	0.65 (0.09, 5.24)	2.62 (0.23, 26.81)	2.74 (0.21, 34.6)	2.17 (0.16, 26.69)	BT

Acu, acupuncture; HM, herbal medicine; PBNT, Plum Blossom Needle Tapping; WM, Western medicine.

According to Table 11, the best intervention for acupuncture therapies to reduce headache frequency may be acupuncture + herbal medicine. The probability sorting histogram is shown in Supplementary Figure 3.

**Table 11:** SUCRA values of headache frequency and ranking of interventions.

**Rank**	**SUCRA**	**Treatments**
1	0.76496	Acu + HM
2	0.76475	Acu + WM
3	0.70009	PBNT
4	0.42981	BT
5	0.25476	Acu
6	0.08563	WM

Acu, acupuncture; HM, herbal medicine; PBNT, Plum Blossom Needle Tapping; WM, Western medicine.

### 3.6. Adverse events

Three out of thirty studies reported the occurrence of adverse events. Zhou (44) reported two adverse reactions, nausea and vomiting, and local skin allergy. The incidence of adverse reactions was 4.41 and 2.94%, respectively. Wu and Sun (40) reported four kinds of adverse reactions: nausea and vomiting, fever, local skin allergy, and systemic reaction; the incidence of adverse events was 18.50%. Guo (21) reported the recurrence of some cases during treatment and retching and fatigue during follow-up, all of which occurred in the control group. We found that the most common adverse reactions may be local skin allergies and nausea and vomiting caused by needle fainting or other reasons. Based on the current studies, no serious adverse events have been found yet.

### 3.7. Publication bias

By testing for publication bias for total effective rate and VAS, we found that studies reporting total effective rate were less likely to have publication bias because these points were concentrated in the interior of the triangle and distributed on both sides of the midline (Supplementary Figure 4A); however, studies reporting VAS may have some risk of bias or small sample effects (Supplementary Figure 4B).

## 4. Discussion

The third edition of the International Headache Society classification of headache disorders divides TTH into four categories, Infrequent episodic tension-type headache, Frequent tension-type headache, Chronic tension-type headache, and Probable tension-type headache (3). Among them, frequent TTH and chronic TTH are more common. If the pain time of frequent TTH exceeds 15 days per month, it will turn into chronic TTH. Because some RCTs included in this study did not mention the definite classification of TTH, we did not analyze TTH with different syndromes.

Based on the results of our study, it is evident that the most common control intervention is Western medicine, which is also in line with reality. The treatment of TTH often has pain relief as the primary goal, and analgesics or NSAIDs are commonly used; however, for chronic tension-type headaches, analgesics alone are ineffective, and overdose has the risk of aggravating pain. So people often seek a long-term effective, economically safe complementary or alternative therapy. Therefore, we chose acupuncture therapy as the focus of this study. According to a previous Cochrane analysis, acupuncture is valuable in treating TTH (12, 13). Due to the wide variety of acupuncture therapies, exploring which treatment or combination is the most effective became our research objective. Through a pre-developed study plan and a comprehensive search and screening of the literature on acupuncture therapies for TTH, we ultimately identified 30 RCTs that met the inclusion criteria. We summarized the types of acupuncture interventions in 30 RCTs: conventional acupuncture, head acupuncture, bloodletting therapy, Plum Blossom Needle Tapping, and electroacupuncture (or acupuncture combined with other treatments). We analyzed four outcome indicators, total effective rate, VAS, headache frequency, and adverse events, to provide direct and indirect evidence for the optimal protocol of acupuncture-related therapies for TTH. Direct evidence about total effective rate showed that conventional acupuncture, acupuncture + Western medicine, acupuncture + herbal medicine, head acupuncture, head acupuncture + Western medicine, Plum Blossom Needle Tapping, and electroacupuncture + cupping were superior to Western medicine; for VAS scores, conventional acupuncture, acupuncture + Western medicine, and head acupuncture + Western medicine were more effective than Western medicine; conventional acupuncture, acupuncture + Western medicine, and bloodletting were superior to Western medicine for headache frequency. However, some results were heterogeneous (*I*^2^ > 50%). By examining the original RCTs, we suggest that the heterogeneity may come from the clinical design, including the selection of acupuncture points, depth, duration of acupuncture, and the choice of drugs in the control group. When conducting NMA, the results of the ranked probability of the total effective rate of acupuncture therapies on TTH showed that bloodletting therapy had the highest probability of being the best intervention (SUCRA value = 0.93156136); for pain level, we found that head acupuncture + Western medicine was effective in reducing VAS scores compared to other interventions, in addition, acupuncture + herbal medicine may be the best therapy for reduce TTH pain frequency. However, we found no statistically significant between the comparison of different interventions after combining direct and indirect evidence. This finding should be interpreted cautiously due to the considerable heterogeneity of results. We attempted to explore the influence of acupuncture therapies on headache duration. Unfortunately, the time measurement unit was not consistent in these RCTs. Therefore, this study did not perform an analysis of headache duration. Although the adverse events caused by acupuncture were mild (46), several were reported in some studies, with a high incidence of the following 2, gastrointestinal discomfort (mainly manifested as nausea and vomiting) and localized skin sensitivity. These two adverse events may be closely related to pre-needle communication and standardization of the procedure. For the needle-shy population, proper communication before an acupuncture operation, introducing the patient to the acupuncture process, and explaining the possible normal reactions can help to eliminate the patient's nervousness and thus reduce the occurrence of adverse events.

Studies have shown that the pathogenesis of TTH can be divided into four categories: myofascial, vascular, central, and genetic mechanisms (47). Myofascial mechanisms suggest that TTH is caused by localized strain, inflammation, and ischemia in the head, neck, and shoulder muscle fiber bands. Pain is triggered by the sensitization of pain receptors caused by sustained muscle contraction and afferent myofascial nociceptive receptors, which may result from the release of endogenous inflammatory mediators, such as IL-6, bradykinin, and serotonin, in the blood (48). Vascular mechanisms indicate that the increased blood flow velocity in the basilar artery of the brain in patients with TTH supports the idea that TTH may occur due to abnormal blood flow in intracranial vessels. Calcitonin gene-related peptide and nitric oxide (NO) may cause abnormal blood flow in TTH. Prolonged stress in TTH patients will lead to the production of large amounts of free radicals. Superoxide dismutase (SOD) can scavenge free radicals in the body in time to protect the organism from damage, while repeated episodes of TTH make SOD depleted. Its activity is reduced, severely weakening the protective effect on vascular endothelium. The damaged vascular endothelium can release large amounts of NO, which dilates intracranial blood vessels and causes pain (49, 50). Central mechanisms dominate chronic TTH; the core mechanism is central sensitization related to NO. Studies have found increased nitric oxide synthase (NOS) activity in platelets and decreased levels of 5-hydroxytryptamine (5-HT) in patients with TTH. They reflected pain modulation in the spinal cord and trigeminal nucleus (51). Animal experiments have shown that needling myofascial trigger points reduce substance P and increase levels of endogenous opioids, such as spinal enkephalin and serum β-endorphin, thereby providing an analgesic effect (52, 53). This is also a potential analgesic mechanism for myofascial pain management. In addition, it has also been found that in an animal model of spinal cord injury-induced neurogenic pain, acupuncture can relieve pain by inhibiting the inflammatory response of activated microglia MAPKs after spinal cord injury, suggesting that acupuncture can control the central inflammatory response by inhibiting the activation of central microglia, thereby reducing central sensitization for analgesia (54). Similarly, studies have shown that electroacupuncture can accelerate vertebral artery blood flow, improve blood circulation in the vertebrobasilar system, increase blood and oxygen supply to brain tissue, improve cerebral circulation, and regulate serum NO levels, thus providing analgesia (55). Bloodletting therapy is one of the characteristic therapies of Traditional Chinese Medicine and an essential branch of acupuncture. The mechanism of bloodletting therapy for TTH is still unclear. The available studies suggest that bloodletting therapy can stimulate local nerves and cause central specific nucleus activation, which can play a role in regulating vasoconstriction to a certain extent (56). The above summary provides the theoretical basis for our findings and a reference for subsequent studies.

### 4.1. Strengths

As the first study to explore the effectiveness and safety of different acupuncture therapies for TTH, we used broad and rigorous inclusion and exclusion criteria and detailed search strategies. We conducted a comprehensive search of nine databases. Before our study, we developed a detailed study protocol and registered it in PROSPERO to ensure transparency and openness of the study. We first conducted a pairwise meta-analysis and then NMA to synthesize direct and indirect evidence for the results. In addition, due to the closed loop formed in the network evidence plot, we performed an inconsistency analysis. The results showed that the consistency of the direct and indirect comparison results was good (*p* > 0.05). The convergence diagnostic plots show that the convergence of the model was good, indicating that our results are reliable.

### 4.2. Limitations

First, most of the RCTs were of average quality, limiting the credibility of the conclusions. Second, all RCTs were conducted in China. Some did not have detailed disease classifications, so we did not analyze TTH separately for different types. Third, we found no uniform drug for the control group. The same acupuncture type was not standardized for acupuncture points, angle, depth, and retention time, all of which contributed to the heterogeneity of the results. Fourth, most studies lacked follow-up reports, so long-term efficacy was not verified. Fifth, for safety, only three studies reported adverse events, all of which were descriptive, so it is difficult to analyze the safety of acupuncture therapy for TTH quantitatively.

### 4.3. Future prospects

First, future RCTs should strictly follow the Standards for Reporting Interventions in Controlled Trials of Acupuncture (STRICTA) and be conducted in many countries to improve the quality of evidence. Secondly, the classification of TTH must be carried out in strict accordance with the diagnostic criteria of the International Headache Society to provide personalized treatment programs and reduce the heterogeneity of results. Thirdly, the existing studies lack standardized and unified evaluation criteria for curative effects. The outcome indicators should be unified based on the precise classification in the future. Fourthly, long-term efficacy evaluation should be added to provide treatment ideas for chronic TTH. Fifthly, it is necessary to pay attention to the safety of acupuncture therapy in treating TTH and fully report the incidence of adverse events to provide reliable data for safety evaluation.

## 5. Conclusion

Our findings suggest that acupuncture therapies for TTH are promising with mild adverse reactions. Among them, bloodletting is expected to be the best intervention to improve the overall symptoms of TTH; for relieving the degree of headache, head acupuncture combined with Western medicine may be the most effective combination therapy; acupuncture combined with herbal medicine seems to reduce the frequency of headaches, but this result should be interpreted with caution.

## Data availability statement

The datasets presented in this study can be found in online repositories. The names of the repository/repositories and accession number(s) can be found in the article/Supplementary material.

## Author contributions

JH: research design and writing. XW: statistical guidance and manuscript revision. YW and XX: literature screening and data extraction. LK and SJ: literature quality assessment. YH and XC: manuscript revision. All authors contributed to this study and approved the final manuscript.
