# Supplementary material for: Acupuncture and related therapies for tension-type headache: a systematic review and network meta-analysis
Source: Front Neurol. 2023 Jun 22;14:1194441. doi: 10.3389/fneur.2023.1194441 (PMC10325685; doi:10.3389/fneur.2023.1194441)
Supplement: Supplementary file 1 [file Data_Sheet_1.docx]

**Box. Terminology: Reviews With Networks of Multiple Treatments**

Different terms have been used to identify systematic reviews that incorporate a network of multiple treatment comparisons. A brief overview of common terms follows.

*Indirect treatment comparison:* Comparison of 2 interventions for which studies against a common comparator, such as placebo or a standard treatment, are available (i.e., indirect information). The direct treatment effects of each intervention against the common comparator (i.e., treatment effects from a comparison of interventions made within a study) may be used to estimate an indirect treatment comparison between the 2 interventions (**Appendix Figure 1, A**). An indirect treatment comparison (ITC) may also involve multiple links. For example, in **Appendix Figure 1, B**, treatments B and D may be compared indirectly on the basis of studies encompassing comparisons of B versus C, A versus C, and A versus D.

*Network meta-analysis* or *mixed treatment comparison*: These terms, which are often used interchangeably, refer to situations involving the simultaneous comparison of 3 or more interventions. Any network of treatments consisting of strictly unclosed loops can be thought of as a series of ITCs (**Appendix Figure 1, A and B**). In mixed treatment comparisons, both direct and indirect information is available to inform the effect size estimates for at least some of the comparisons; visually, this is shown by closed loops in a network graph (**Appendix Figure 1, C**). Closed loops are not required to be present for every comparison under study. "Network meta-analysis" is an inclusive term that incorporates the scenarios of both indirect and mixed treatment comparisons.

*Network geometry evaluation:* The description of characteristics of the network of interventions, which may include use of numerical summary statistics. This does not involve quantitative synthesis to compare treatments. This evaluation describes the current evidence available for the competing interventions to identify gaps and potential bias. Network geometry is described further in **Appendix Box 4**.

**Appendix Box 1. The Assumption of Transitivity for Network Meta-Analysis**

Methods for indirect treatment comparisons and network meta-analysis enable learning about the relative treatment effects of, for example, treatments A and B through use of studies where these interventions are compared against a common therapy, C.

When planning a network meta-analysis, it is important to assess patient and study characteristics across the studies that compare pairs of treatments. These characteristics are commonly referred to as *effect modifiers* and include traits such as average patient age, gender distribution, disease severity, and a wide range of other plausible features.

For network meta-analysis to produce valid results, it is important that the distribution of effect modifiers is similar, for example, across studies of A versus B and A versus C. This balance increases the plausibility of reliable findings from an indirect comparison of B versus C through the common comparator A. When this balance is present, the assumption of transitivity can be judged to hold.

Authors of network meta-analyses should present systematic (and even tabulated) information regarding patient and study characteristics whenever available. This information helps readers to empirically evaluate the validity of the assumption of transitivity by reviewing the distribution of potential effect modifiers across trials.

**Appendix Box 2. Differences in Approach to Fitting Network Meta-Analyses**

Network meta-analysis can be performed within either a frequentist or a Bayesian framework. Frequentist and Bayesian approaches to statistics differ in their definitions of probability. Thus far, the majority of published network meta-analyses have used a Bayesian approach.

Bayesian analyses return the posterior probability distribution of all the model parameters given the data and prior beliefs (e.g., from external information) about the values of the parameters. They fully encapsulate the uncertainty in the parameter of interest and thus can make direct probability statements about these parameters (e.g., the probability that one intervention is superior to another).

Frequentist analyses calculate the probability that the observed data would have occurred under their sampling distribution for hypothesized values of the parameters. This approach to parameter estimation is more indirect than the Bayesian approach.

Bayesian methods have been criticized for their perceived complexity and the potential for subjectivity to be introduced by choice of a prior distribution that may affect study findings. Others argue that explicit use of a prior distribution makes transparent how individuals can interpret the same data differently. Despite these challenges, Bayesian methods offer considerable flexibility for statistical modeling.

In-depth introductions to Bayesian methods and discussion of these and other issues can be found elsewhere.

**Appendix Box 3. Network Meta-Analysis and Assessment of Consistency**

Network meta-analysis often involves the combination of direct and indirect evidence. In the simplest case, we wish to compare treatments A and B and have 2 sources of information: direct evidence via studies comparing A versus B, and indirect evidence via groups of studies comparing A and B with a common intervention, C. Together, this evidence forms a closed loop, ABC.

Direct and indirect evidence for a comparison of interventions should be combined only when their findings are similar in magnitude and interpretation. For example, for a comparison of mortality rates between A and B, an odds ratio determined from studies of A versus B should be similar to the odds ratio comparing A versus B estimated indirectly based on studies of A versus C and B versus C. This assumption of comparability of direct and indirect evidence is referred to as *consistency* of treatment effects.

When a treatment network contains a closed loop of interventions, it is possible to examine statistically whether there is agreement between the direct and indirect estimates of intervention effect.

Different methods to evaluate potential differences in relative treatment effects estimated by direct and indirect comparisons are grouped as *local approaches* and *global approaches.* Local approaches (e.g., the Bucher method or the node-splitting method) assess the presence of inconsistency for a particular pairwise comparison in the network, whereas global approaches (e.g., inconsistency models, *I*^2^ measure for inconsistency) consider the potential for inconsistency in the network as a whole.

Tests for inconsistency can have limited power to detect a true difference between direct and indirect evidence. When multiple loops are being tested for inconsistency, one or a few may show inconsistency simply by chance. Further discussions of consistency and related concepts are available elsewhere.

Inconsistency in a treatment network can indicate lack of transitivity (see **Appendix Box 1**).

**Appendix Box 4. Network Geometry and Considerations for Bias**

The term *network geometry* is used to refer to the architecture of the treatment comparisons that have been made for the condition under study. This includes what treatments are involved in the comparisons in a network, in what abundance they are present, the respective numbers of patients randomly assigned to each treatment, and whether particular treatments and comparisons may have been preferred or avoided.

Networks may take on different shapes. Poorly connected networks depend extensively on indirect comparisons. Meta-analyses of such networks may be less reliable than those from networks where most treatments have been compared against each other.

Qualitative description of network geometry should be provided and accompanied by a network graph. Quantitative metrics assessing features of network geometry, such as *diversity* (related to the number of treatments assessed and the balance of evidence among them), *co-occurrence* (related to whether comparisons between certain treatments are more or less common), and *homophily* (related to the extent of comparisons between treatments in the same class versus competing classes), can also be mentioned.

Although common, established steps for reviewing network geometry do not yet exist, however examples of in-depth evaluations have been described related to treatments for tropical diseases and basal cell carcinoma and may be of interest to readers. An example based on 75 trials of treatments for pulmonary arterial hypertension (**Appendix Figure 3**) suggests that head-to-head studies of active therapies may prove useful to further strengthen confidence in interpretation of summary estimates of treatment comparisons.

**Appendix Box 5. Probabilities and Rankings in Network Meta-Analysis**

Systematic reviews incorporating network meta-analyses can provide information about the hierarchy of competing interventions in terms of treatment rankings.

The term *treatment ranking probabilities* refers to the probabilities estimated for each treatment in a network of achieving a particular placement in an ordering of treatment effects from best to worst. A network of 10 treatments provides a total of 100 ranking probabilities—that is, for each intervention, the chance of being ranked first, second, third, fourth, fifth, and so forth).

Several techniques are feasible to summarize relative rankings, and include graphical tools as well as different approaches for estimating ranking probabilities. **Appendix Figure 6** shows 2 approaches to presenting such information, on the basis of a comparison of adjuvant interventions for resected pancreatic adenocarcinoma.

Robust reporting of rankings also includes specifying median ranks with uncertainty intervals, cumulative probability curves, and the surface under the cumulative ranking (SUCRA) curve.

Rankings can be reported along with corresponding estimates of pairwise comparisons between interventions. Rankings should be reported with probability estimates to minimize misinterpretation from focusing too much on the most likely rank.

Rankings may exaggerate small differences in relative effects, especially if they are based on limited information. An objective assessment of the strength of information in the network and the magnitude of absolute benefits should accompany rankings to minimize potential biases.

**Appendix Figure 1A-1C**


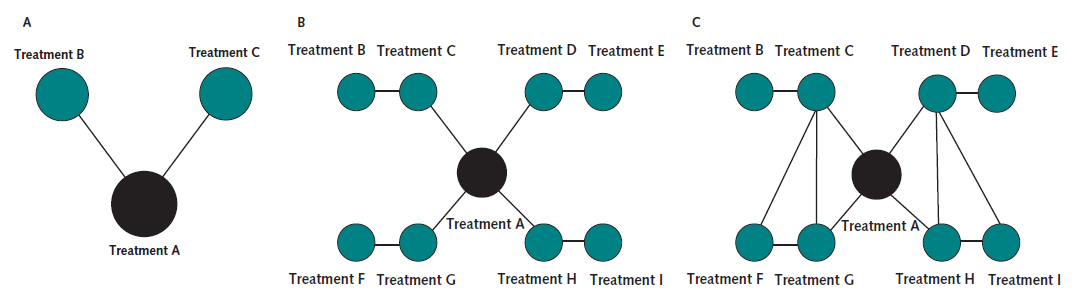


**Appendix Figure 3**


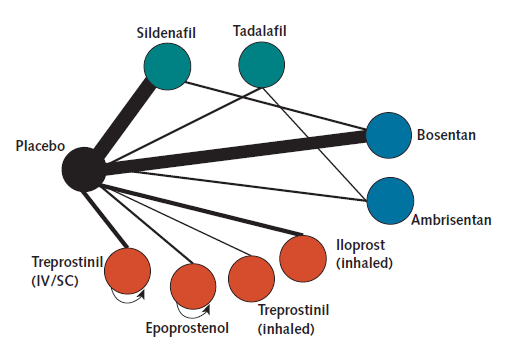


**Appendix Figure 6**

**
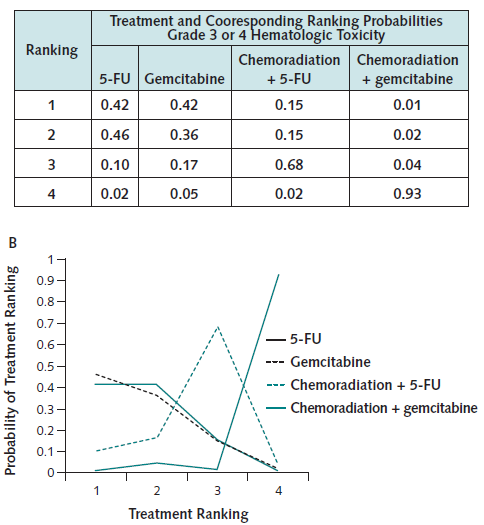
**
