# Supplementary figures and images for: Acupuncture and related therapies for tension-type headache: a systematic review and network meta-analysis
Source: Front Neurol. 2023 Jun 22;14:1194441. doi: 10.3389/fneur.2023.1194441 (PMC10325685; doi:10.3389/fneur.2023.1194441)

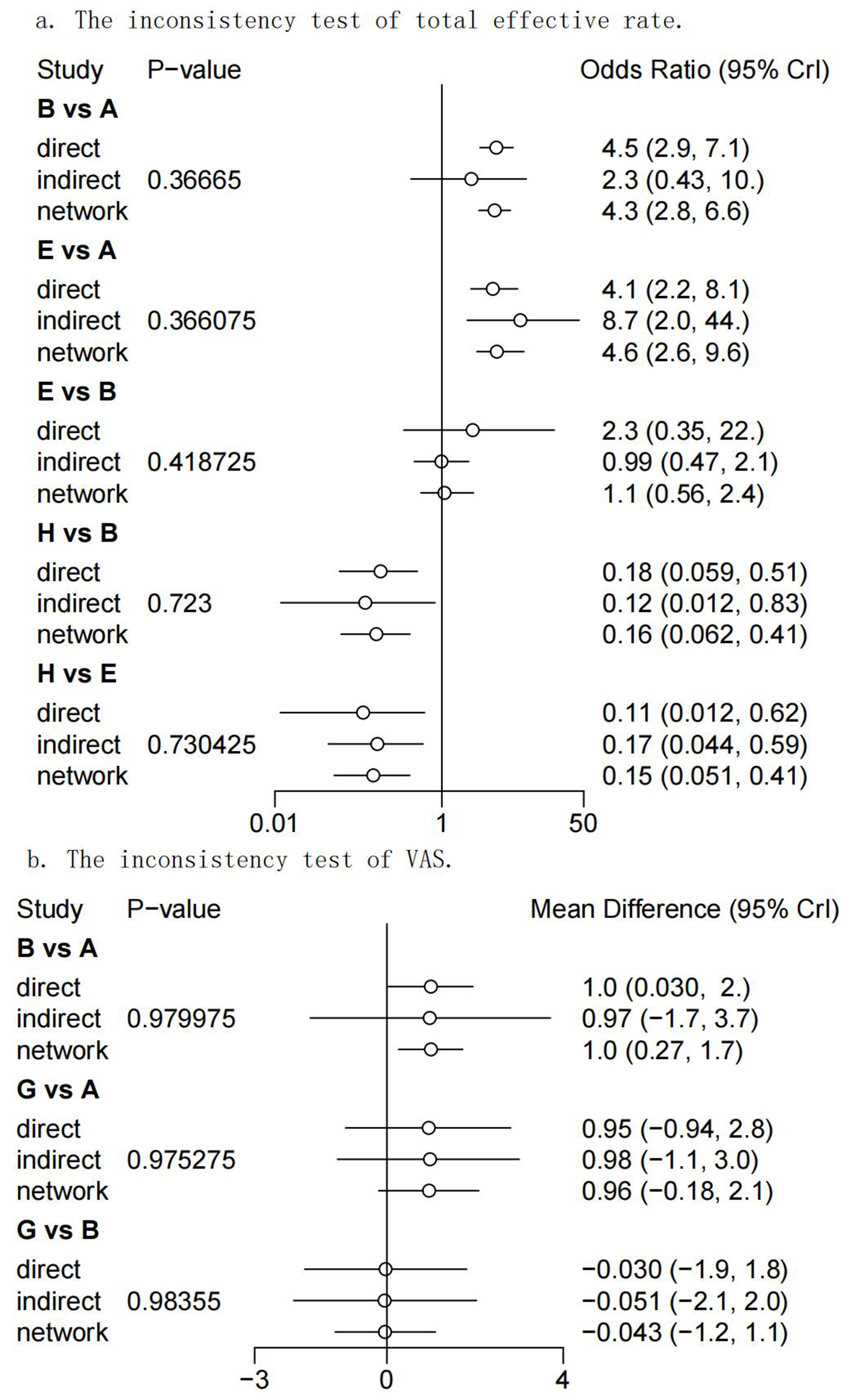

Supplement: Supplementary file 2 [file Image_1.JPEG]

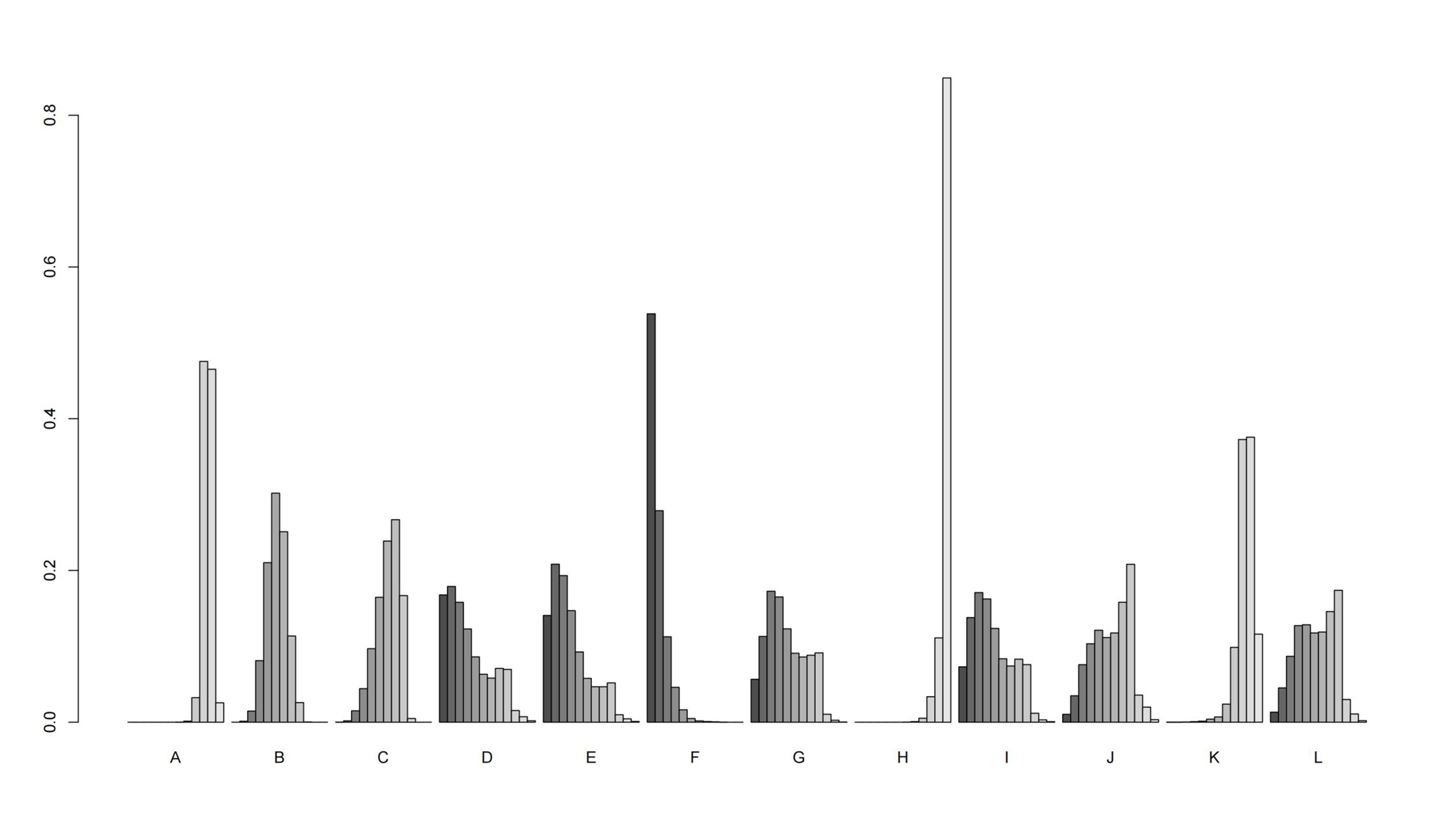

Supplement: Supplementary file 3 [file Image_2.JPEG]

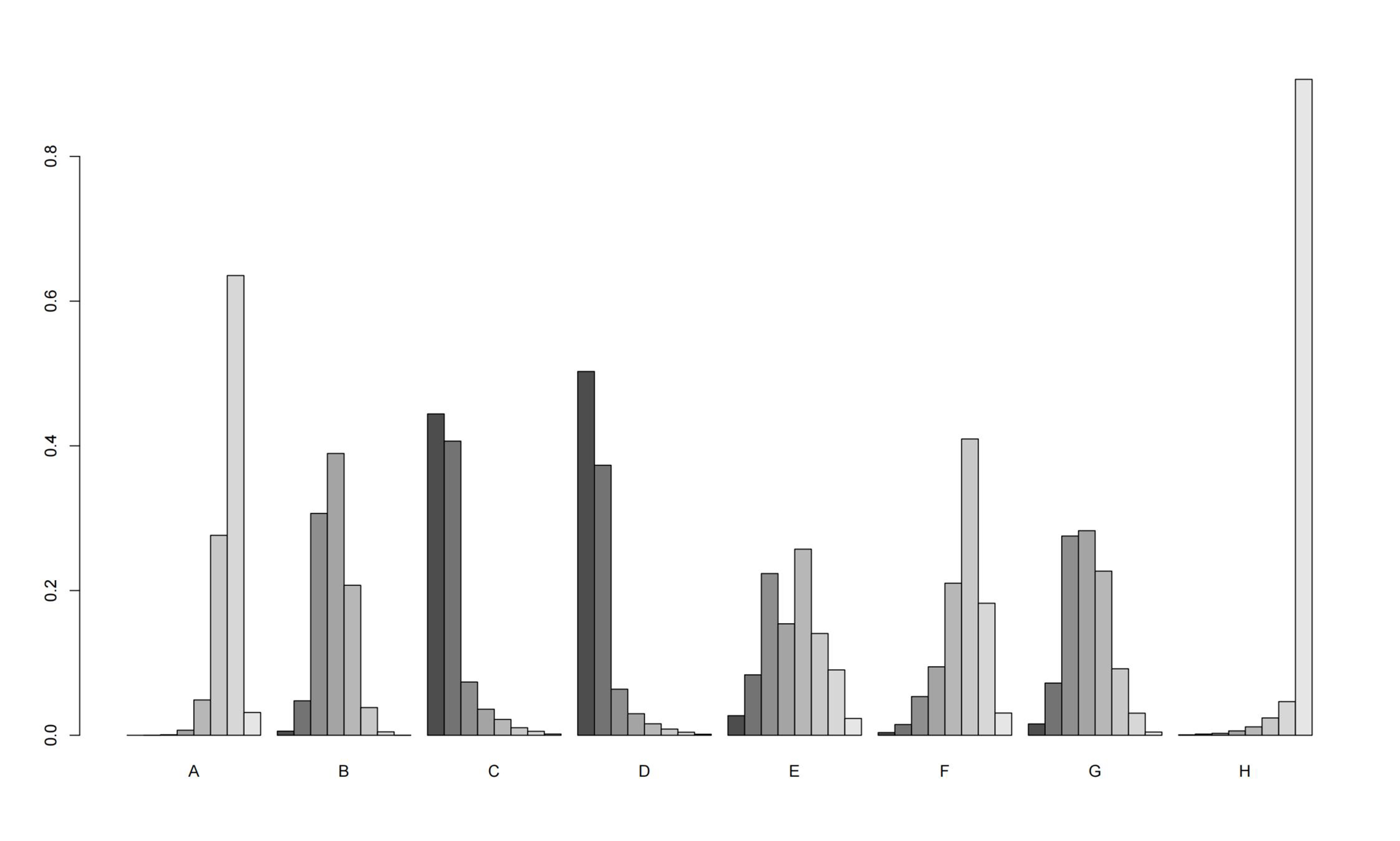

Supplement: Supplementary file 4 [file Image_3.JPEG]

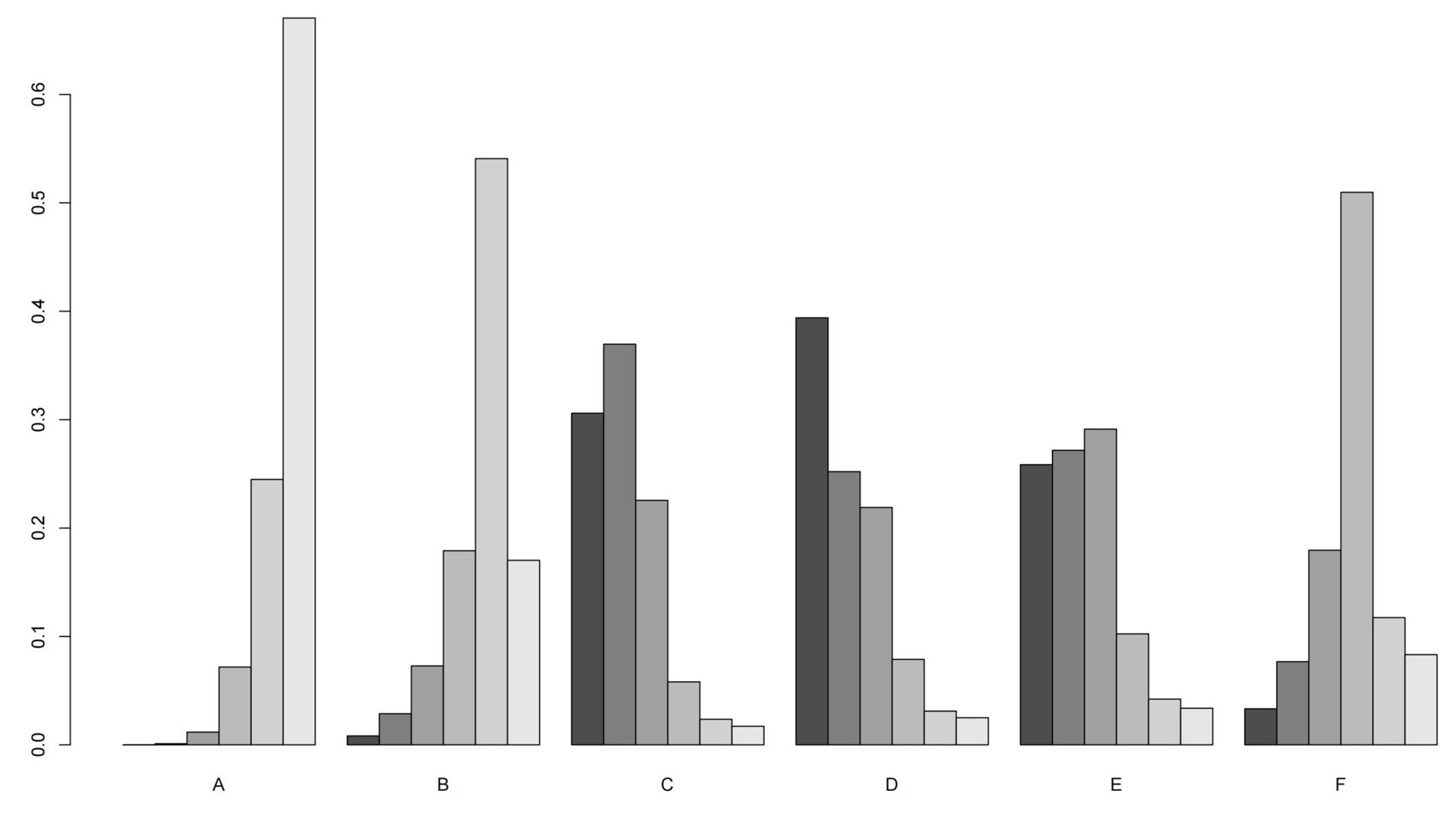

Supplement: Supplementary file 5 [file Image_4.JPEG]

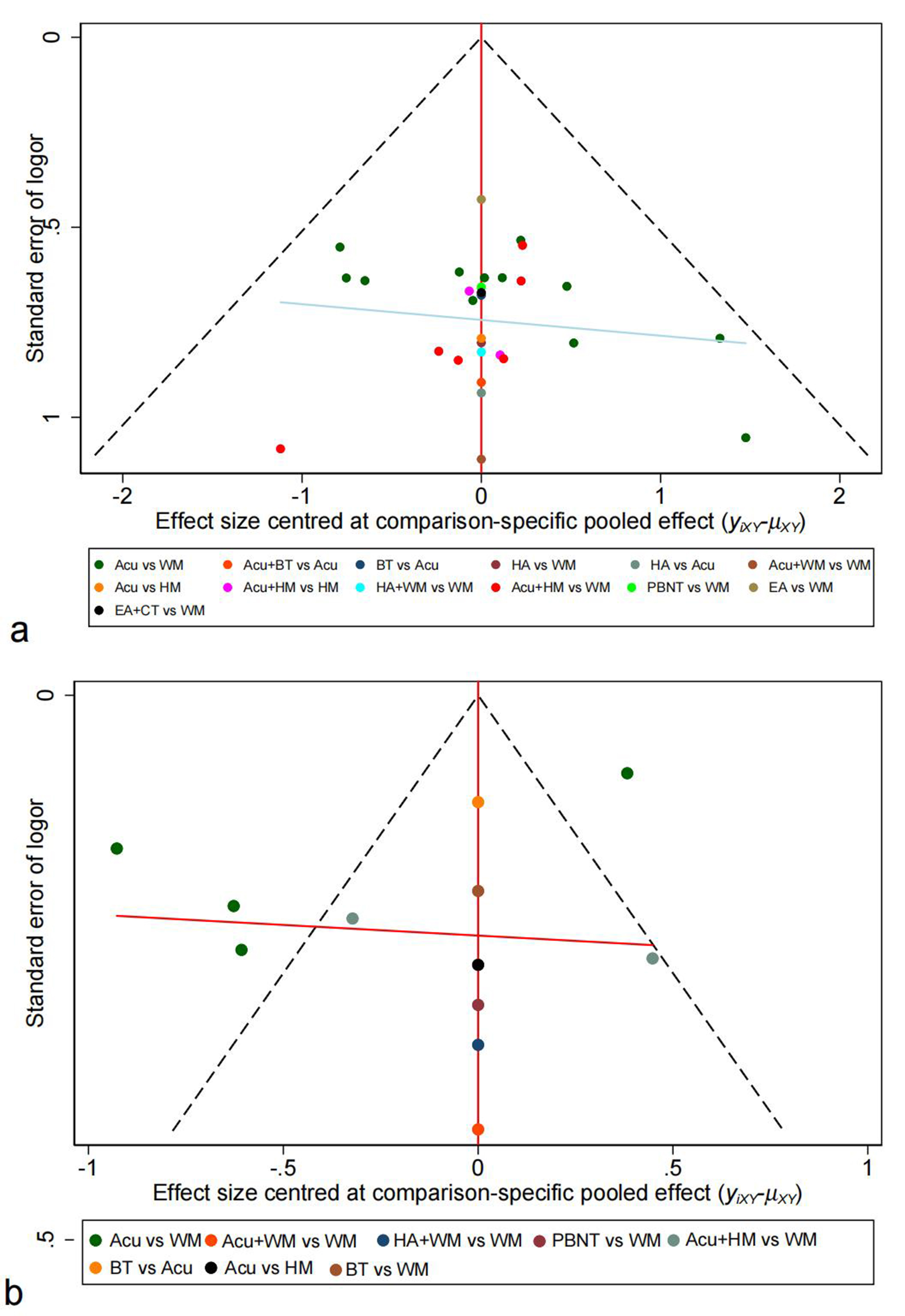

Supplement: Supplementary file 6 [file Image_5.JPEG]
